# Maternal Short-Chain Fructooligosaccharide Supplementation Influences Intestinal Immune System Maturation in Piglets

**DOI:** 10.1371/journal.pone.0107508

**Published:** 2014-09-19

**Authors:** Cindy Le Bourgot, Stéphanie Ferret-Bernard, Laurence Le Normand, Gérard Savary, Enrique Menendez-Aparicio, Sophie Blat, Emmanuelle Appert-Bossard, Frédérique Respondek, Isabelle Le Huërou-Luron

**Affiliations:** 1 INRA UR1341 ADNC, Saint-Gilles, France; 2 Tereos-Syral, Markolsheim, France; National Institute of Agronomic Research, France

## Abstract

*Peripartum* nutrition is crucial for developing the immune system of neonates. We hypothesized that maternal short-chain fructooligosaccharide (scFOS) supplementation could accelerate the development of intestinal immunity in offspring. Thirty-four sows received a standard or a scFOS supplemented diet (10 g scFOS/d) for the last 4 weeks of gestation and the 4 weeks of lactation. Colostrum and milk immunoglobulins (Ig) and TGFβ1 concentrations were evaluated on the day of delivery and at d 6 and d 21 postpartum. Piglet intestinal structure, the immunologic features of jejunal and ileal Peyer's patches, and mesenteric lymph node cells were analysed at postnatal d 21. Short-chain fatty acid concentrations were measured over time in the intestinal contents of suckling and weaned piglets. Colostral IgA (*P*<0.05) significantly increased because of scFOS and TGFβ1 concentrations tended to improve (*P*<0.1). IFNγ secretion by stimulated Peyer's patch and mesenteric lymph node cells, and secretory IgA production by unstimulated Peyer's patch cells were increased (*P*<0.05) in postnatal d 21 scFOS piglets. These differences were associated with a higher proportion of activated CD25+CD4α+ T cells among the CD4+ helper T lymphocytes (*P*<0.05) as assessed by flow cytometry. IFNγ secretion was positively correlated with the population of activated T lymphocytes (*P*<0.05). Total short-chain fatty acids were unchanged between groups during lactation but were higher in caecal contents of d 90 scFOS piglets (*P*<0.05); specifically propionate, butyrate and valerate. In conclusion, we demonstrated that maternal scFOS supplementation modified the intestinal immune functions in piglets in association with increased colostral immunity. Such results underline the key role of maternal nutrition in supporting the postnatal development of mucosal immunity.

## Introduction

Prenatal and early postnatal life represents a crucial period during which many systems develop, such as the gut immune system, and commensal microbiota. As in other species, the immune system is functionally immature at birth in the neonatal piglet containing poorly developed gut-associated lymphoid tissue (GALT), unlike systemic immunity [1]. Contact of GALT with the environment and colonizing bacteria from birth is essential for healthy immune maturation and the development of tolerance [2]. Breastfeeding also exerts a major influence in early infancy by modulating the maturation of the neonatal immune system. Due to the presence of immunological factors (IgG, IgA, IgM and cytokines), colostrum and breast milk represent a crucial source of passive immunity [3]. Secretory IgA (sIgA) resists digestion in the gut, where it binds to enteric pathogens to protect the suckling neonate. IgG transferred from sow serum to mammary secretions give added protection against pathogenic species [4]. Colostrum, and to a lesser extent milk, also contain immunosuppressive cytokines such as TGFβ1 and IL-10, which participate in inducing tolerance to harmless food antigens and commensal bacteria [5]. Finally, environmental antigens from the mother's intestine, transported to the mammary gland, are transferred *via* colostrum and milk to the neonate and affect its immune responses [6], [7].

Prebiotics are defined as selectively-fermented ingredients that result in specific changes in the gut microbiome and the production of bacterial metabolites which contribute to their beneficial properties for the host [8], [9]. Short-chain fructooligosaccharides (scFOS) obtained from sucrose belong to the wide family of β-fructans and are well-known prebiotics. They were first associated with a specific stimulation of bifidobacteria and lactobacilli in infants or adults, and more recently with changes in *Clostridium coccoides* [10], [11], [12], [13], [14]. Bifidobacteria and lactobacilli primarily produce acetate and lactate as fermentation metabolites. Recent studies provide a better understanding of the butyrogenic effect of scFOS. Indeed, functional metagenomic analysis highlighted novel prebiotic degradation pathways and revealed effects of scFOS on carbohydrate transporters and hydrolysis enzymes in bacteria such as *Bifidobacterium longum, Dorea longicatena, Eubacterium rectale* and in as yet unidentified bacterial groups [15]. Moreover it has been hypothesised that butyrate-producing bacteria in the colon could cause a butyrogenic effect by cross-feeding interactions rather than by FOS consumption directly [16]. Prebiotic ingestion results in trophic effects on the distal intestine and higher GALT proliferation [17]. In adults, direct FOS supplementation increases the development of lymph nodes producing higher amounts of IFNγ, and promotes intestinal IgA secretion [18], [19]. This direct effect on sIgA has also been substantiated in formula-fed infants supplemented with prebiotics [20], [21]. The addition of prebiotics to the maternal diet during pregnancy has been shown to change the intestinal microbiota composition of both the mother and the offspring [22], [23], [24] confirming the existence of a microbiota transfer from the mother to the newborn [25]. Intestinal microbiota establishment plays a crucial role in GALT proliferation and maturation as well as in the recruitment of IgA-secreting plasma cells and T cells to mucosal sites. Microbiota-derived signals influence the crosstalk between epithelial cells and gut dendritic cells, thereby modulating the nature and intensity of intestinal B and T cell responses. Moreover, the role of prebiotic supplementation of the maternal diet on lactogenic immunity has been demonstrated in some studies with animal models (*i.e.* increased Ig contents in colostrum and/or mature milk) [26], [27], [28]. However, less is known about the effects of the transfer of these benefits on GALT maturation in offspring.

The objective of this study was to assess the impact of maternal dietary scFOS supplementation given at a physiological dose during gestation and lactation on the developmental profile of the local immune system of suckling offspring in pigs. We hypothesized that maternal dietary scFOS supplementation will modify lactogenic immunity and improve the developmental pattern of GALT in newborn piglets.

## Materials and Methods

### Animals, diets and experimental design

**Figure 1:**
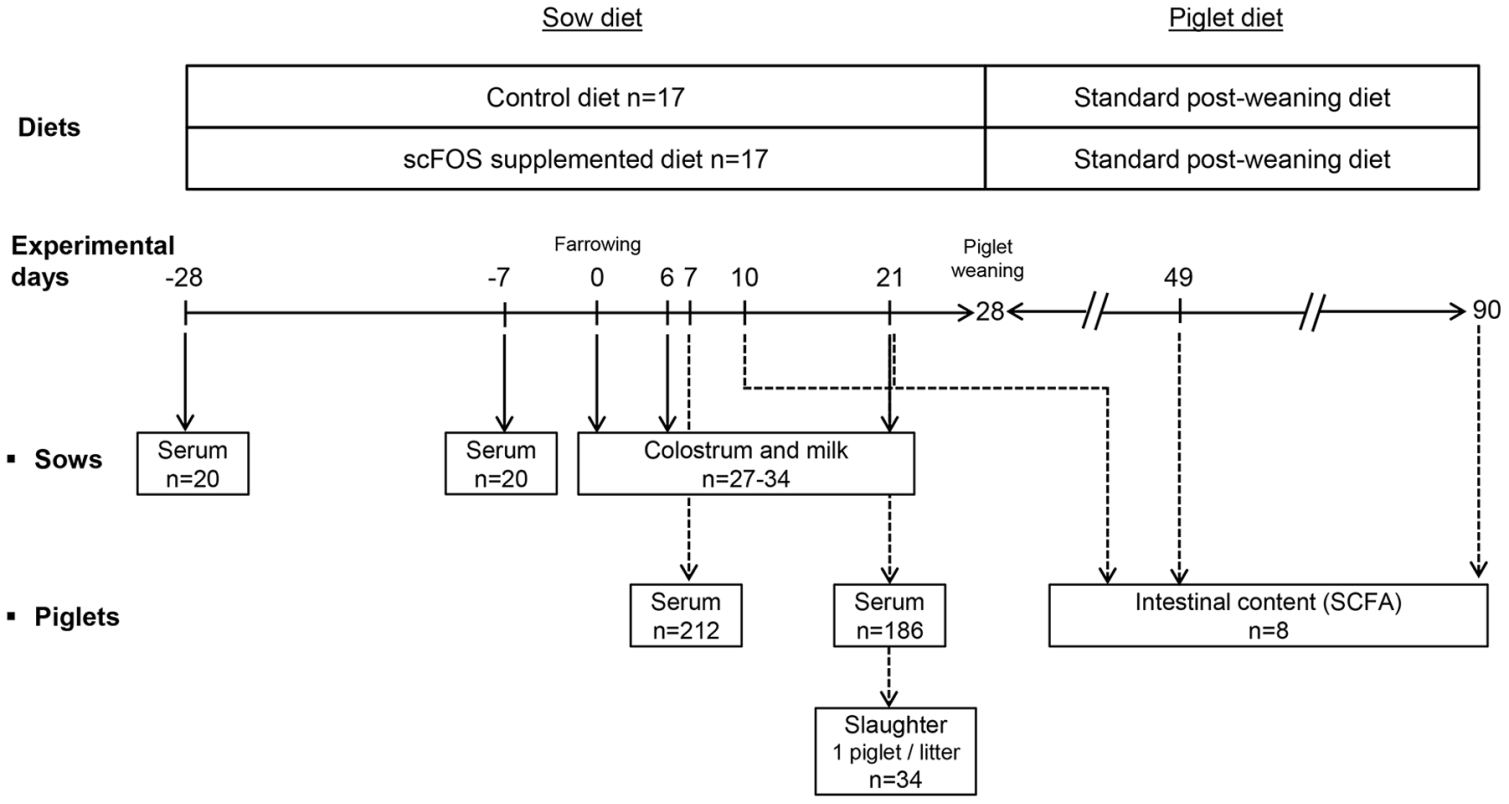
Overview of the study design. Thirty-four sows and their piglets (369 piglets) were used in 4 replications (n = 11, 9, 6 and 8 in the 1^st^ (R1), 2^nd^ (R2), 3^rd^ (R3) and 4^th^ (R4) replications respectively). From 28 d before the expected time of farrowing, sows were fed either a control diet (n = 17) or a short-chain fructooligosaccharides (scFOS; 95% of scFOS with molecular chain length between 3 and 5 monomeric unity, Profeed P95, Beghin-Meiji, Marckolsheim, France)-supplemented diet (n = 17) until the end of the lactation. Serum was collected in gestating sows (n = 20 from R1 and R2) and suckled piglets (n = 212 from R1 and R2 at postnatal d 7; n = 186 from R1 and R2 at postnatal d 21) in order to analyse immunoglobulin concentration. Immunoglobulin and cytokine concentrations were also determined in samples of colostrum (n = 27 from the 4 replications; colostrum was not collected when sows farrowed nightly) and milk (n = 34 from the 4 replications at d 6 and d 21). One piglet per litter was sacrificed at 21 d of age (n = 34 from the 4 replications) to investigate the development of the intestinal immune system. A subset of piglets from R4 (n = 8/age) was used during the suckling period at postnatal d 10 and d 21, and during the post-weaning period at postnatal d 49 and d 90 to perform short-chain fatty acid (SCFA) production in intestinal contents.

The experimental protocol was designed in compliance with legislations of the European Union (directive 86/609/EEC) and France (decree 2001-464 29/05/01) for the care and use of laboratory animals (agreement for animal housing number B-35-275-32 and certificate of authorization number 006061 to experiment on live animals). Thirty-four sows (Large White x Landrace, 241.7±6.4 kg) and their piglets ((Large White x Landrace) x Pietrain) from the INRA experimental herd (Saint-Gilles, France) were used in 4 replications. Animals were observed daily to ensure their welfare. Diets were formulated according to the nutrient and energy requirements of gestating and lactating sows. They were based on regular gestation or lactation diets (Cooperl, Lamballe, France) supplied with either maltodextrin (control group, n = 17; CTRL) or scFOS (95% of scFOS with molecular chain length between 3 and 5 monomeric unity, Beghin-Meiji, Marckolsheim, France; scFOS group, n = 17) **(** **Table 1** **)**. Sows were separated into two groups at the 87^th^ day of gestation. The first group was fed the CTRL diet for the last 4 weeks of gestation and the 4 weeks of lactation while the second group was fed the scFOS diet **(** **Figure 1** **)**. Sows were given 3 kg.day^−1^ of feed during gestation and were fed *ad libitum* during lactation, the transition from 3 kg.day^−1^ to *ad libitum* being gradual within 4 d. Supplementation of the sow diet with 0.33% and 0.15% during gestation and lactation respectively resulted in scFOS intake of approximately 10 g/d. Sow body weight and feed intake were recorded. Their back fat thickness was measured ultrasonically (Sonolayer SAL-32B, Toshiba, Tokyo, Japan) at the P2-position on both sides of the sow 7 days before and 14 and 28 after parturition. Parturitions were not induced. The number of stillborn and born-alive piglets within litter was recorded. In the 12 h following farrowing, the litter size and the individual piglet birth weight were measured. When possible the litter size was adjusted to 11 piglets by adding (when litter size <11 piglets) or removing (when litter size>11 piglets) piglets within each sows' dietary group. This was done on the second day after parturition, without changing the mean litter birth weight. From birth until weaning, piglets were weighed weekly and their mortality rate was recorded. One piglet whose birth weight was close to the mean litter birth weight was selected from each litter. At postnatal d 21 they were stunned by electronarcosis and killed by exsanguination by a qualified staff member **(** **Figure 1** **)**. In order to evaluate the dynamic profile of intestinal bacterial fermentative activity during suckling and after weaning (weaned piglets were fed with a standard post-weaning diet; Cooperl, Lamballe, France), piglets from the 4^th^ replication were slaughtered at either postnatal d 10, or d 21 during the suckling period or at postnatal d 49 or d 90 post-weaning (CTRL group, n = 4; scFOS group n = 4; **Figure 1**).

**Table 1:** Composition of diets.

	CRTL		scFOS	
	Gestation	Lactation	Gestation	Lactation
**Ingredients (g.kg^−1^)**				
Wheat	229	256	229	256
Corn	110	120	110	120
Barley	348	257	348	257
Wheat bran	159	100	159	100
Soybean meal	100	180	100	180
Sugarcane molasses	-	30	-	30
Vegetable (palm) oil	20	20	20	20
Maltodextrin	3.3	1.5	-	-
scFOS	-	-	3.3	1.5
Calcium carbonate	17.3	12.0	17.3	12.0
Dicalcium phosphate	3.0	10.2	3.0	10.2
Sodium chloride	4.5	4.5	4.5	4.5
Trace element and vitamin mix 1	5.0	5.0	5.0	5.0
**Composition (g/100 g)**				
Minerals	5.7	6.1	5.7	6.1
Protein	13.4	16.4	13.4	16.4
Fat	4.1	4.2	4.1	4.2
Fiber	4.4	4.1	4.4	4.1
Starch	42.8	38.9	42.8	38.9

CTRL: control diet; scFOS: maternal diet supplemented with scFOS.

1 This premix (Cooperl, Lamballe, France) supplied per kg of diet: retinol 10000 UI, cholecalciferol 1500 UI, alpha-tocopherol 45 mg, menadione 2 mg, thiamine 2 mg, riboflavin 4 mg, niacin 20 mg, D-pantothenic acid 10.9 mg, pyridoxine 3 mg, D-biotin 0.2 mg, folic acid 3 mg, vitamin B12 20 µg, choline 500 mg, Fe 81.5 mg as ferrous carbonate and sulfate, Cu 10 mg as copper sulfate, Mn 40 mg as manganese oxide, Zn 99.2 mg as zinc oxide, Co 0.1 mg as cobalt carbonate, I 0.6 mg and Se 0.3 mg.

### Sample collection

Twenty mL colostrum samples were collected 1 to 15 h after the first piglet birth and mature milk (20–50 mL) was collected on d 6 and d 21 postpartum **(** **Figure 1** **)**. Injection of oxytocin (2 mL, Laboratoires Biové, Arques, France) was carried out to facilitate milking. Half of the colostrum and milk samples were immediately frozen at −20°C until Ig analysis. The remaining colostrum and milk samples were centrifuged at 400 g for 12 min at 4°C to collect the aqueous phase which was stored at −20°C until TGFβ1 assay.

Blood was collected from the jugular vein on 20 sows at both the 87^th^ and the 108^th^ day of gestation and on 212 piglets at the 7^th^ day and on 186 piglets at the 21^st^ day of lactation **(** **Figure 1** **)**. After the blood had clotted, serum was separated by centrifugation and stored at −20°C for later analysis.

At slaughter at postnatal d 21, after opening the intestinal cavity, mesenteric lymph nodes (MLN), all individual jejunal Peyer's patches (PP) and a 10 cm segment of distal ileum (containing ileal PP) were collected, rinsed with ice-cold Hank's balanced saline solution supplemented with 50 µg/mL gentamicin, 200 UI/mL penicillin, 200 µg/mL streptomycin and 10 mM hepes for mononuclear cell isolation. The number of jejunal PP and the length of jejunal and ileal PP were recorded. For histomorphometry, 5 cm ileal segments were rinsed with cold phosphate buffered saline and fixed in 4% paraformaldehyde for 24 h until serial dehydration in ethanol and embedding in paraffin. Ileal, caecal and colonic digesta were collected, weighed, mixed with 1 mL 0.5% ortho-phosphoric acid solution per g of digesta and stored at −20°C for later short-chain fatty acid (SCFA) analysis.

### Immunoglobulin assay

Serum samples from sows and piglets as well as samples of sow colostrum and milk were evaluated for IgG and IgA antibody levels. In addition, IgA concentration was measured in cultured jejunal and ileal PP cell supernatants. Concentration of Ig was quantified using swine IgG or IgA ELISA Quantitation Kit (Bethyl Laboratories, Montgomery, Texas, USA). Samples were all diluted in tris buffered saline 1% bovine serum albumin 0.05% Tween-20 according to preliminary assays **(Table S1)**.

### Colostral and milk TGFβ1 assay

TGFβ1 concentration of aqueous fractions of colostrum and milk was quantified using TGFβ1 ImmunoAssay System (Promega Corporation, Madison, USA) according to manufacturer's instructions. *In vivo*, TGFβ1 is processed from a latent form to the bioactive form of the protein. Only the bioactive form is immunoreactive and detectable by TGFβ1 antibody. This processing can be performed *in vitro* by acid treatment [29]. Samples of colostrum and milk were finally diluted 400-fold and 15-fold respectively, to perform the assay.

### Histomorphometry

Histological sections (5 µm) from ileal segments were stained with haematoxylin and eosin and then examined under a light microscope (Nikon Eclipse E40, Nikon Instruments, France) and image analysis software (NIS-Elements AR 3.0, Nikon Instruments). Villi height and area, crypt depth and area, and number and area of PP follicles were measured in a minimum of 15 well-oriented crypt-villus units per piglet.

### Cell isolation and in vitro culture

Cells from freshly-removed MLN were isolated as previously described in sheep model [30]. The method of isolation of jejunal and ileal PP cells was adapted from Haverson et al. [31]. Briefly, cells were isolated from freshly-removed intestine that was rinsed in ice-cold Hank's balanced saline solution. After being separated from the remaining epithelial tissue by dissection, PP mucosa was gently scraped with a glass slide. Mucosal cells were mechanically disrupted in ice-cold Hank's balanced saline solution with 100 IU/mL penicillin, 100 µg/mL streptomycin sulphate and 2% foetal calf serum by vortex. After centrifugation at 600 g, 10 min and 4°C, the cell pellet was suspended for 1 h at 37°C in complete RPMI medium supplemented with 100 IU/mL collagenase and 60 IU/mL. Cell suspension was finally purified on a Percoll gradient (40% and 70% Percoll solutions), and centrifuged at 1,500 g, 30 min at room temperature without brake. Mononuclear cells were collected at the interface between 70% and 40% Percoll phases. MLN and PP cells were suspended in complete RPMI 1640 medium (Sigma) supplemented with 10% foetal calf serum, 100 IU/mL penicillin and 100 µg/mL streptomycin sulphate (complete RPMI) to achieve cell concentration of 20×10^6^ cells/mL. Cells were cultured for 72 h at 37°C under an atmosphere containing 5% CO_2_, in unstimulated conditions or in presence of 5 µg/mL concanavalin A (ConA). sIgA production of 7 day cultured PP cells (density of 5×10^6^ cells/mL in 24-well flat-bottomed plates with RPMI complete medium) was assessed as previously described [32], [18]. Culture supernatants of MLN and PP cells were harvested and stored at −20°C until assayed for IgA and cytokine detection.

### Cytokine pattern of MLN and PP cells

Concentrations of IL-10, IFNγ and TNFα were measured in culture supernatants of MLN and PP cells, using capture sandwich ELISA. IL-10 and TNFα were determined using porcine IL-10 or TNFα ELISA kit (R&D Systems, Lille, France) according to the manufacturer's instructions. IFNγ was quantified by porcine IFNγ Immunoassay kit (R&D System). Cytokine concentrations were given as levels obtained in stimulated condition subtracted from levels in basal condition.

### Flow cytometry analysis

Flow cytometry analysis was performed on ileal PP using specific monoclonal antibodies (mAbs) or isotype-matched mAb (as controls) **(Table S2)**. Cells were incubated with primary mAbs (20 min, 4°C) recognizing porcine surface antigens: Ig κ light chain, CD2α, CD11R1, CD16 and CD25 were all purchased from AbD Serotec (Colmar, France). MAbs against MHC class II, CD4α, CD14, CD172α and TCR1 γ chain specific were from Washington State University (Monoclonal Antibody Center, Pullman, USA) and CD21 was purchased from Clinisciences (Nanterre, France). Ileal PP cells were finally incubated with fluorochrome anti-mouse/rat Ig isotype antibodies (20 min, 4°C) except for labelling of pig CD8α (BD Biosciences, le Pont de Claix, France) and mouse/rat FoxP3 (eBiosciences, Paris, France) that were directly conjugated. Staining of intracellular FoxP3 was performed using the BD Cytofix/Cytoperm fixation and permeabilization kit (BD Biosciences). Finally, cells were analysed with a cell cytometry analyser (Miltenyi Biotech, Paris, France) equipped with a quantification software.

### Haptoglobin assay

Plasma haptoglobin concentration was assayed according to manufacturer's instructions (Haptoglobin, ABCYS SA, Paris, France) using an automated analyser (Konelab 20i, Thermo Fisher Scientific, Cergy Pontoise, France).

### SCFA assay

After centrifugation at 1700 g for 15 min at 4°C, supernatants of intestinal contents diluted with ortho-phosphoric acid solution were stored at −20°C until SCFA assay by gas chromatography [33].

### Statistical analyses

Sow and piglet performances, colostral and milk immune quality were subjected to ANOVA using the General Linear Model of Statistical Analysis Systems software (SAS Institute, Cary, NC) testing diet (CTRL vs. scFOS), parity class and replication effects as the main factors. Parity was categorised in 3 classes: 1 to 2, 3 to 4, 5 or more. Values of immunologic parameters were analysed for diet effect using student test. Finally two-way ANOVA was used to test the effect of diet, age and the interaction between diet and age for SCFA concentrations. When diet effect or interaction between diet and age were significant, differences between dietary groups at each age were further analysed by the Bonferroni's multiple comparison test. Correlation between CD25+CD4α+ T cells and IFNγ secretion was evaluated using Pearson's R-Test. Data were shown as means ± SEM. Differences between groups were considered significant at *P*≤0.05 and tendency when 0.05<*P≤*0.1.

## Results

### Sow and piglet performance

**Figure 2:**
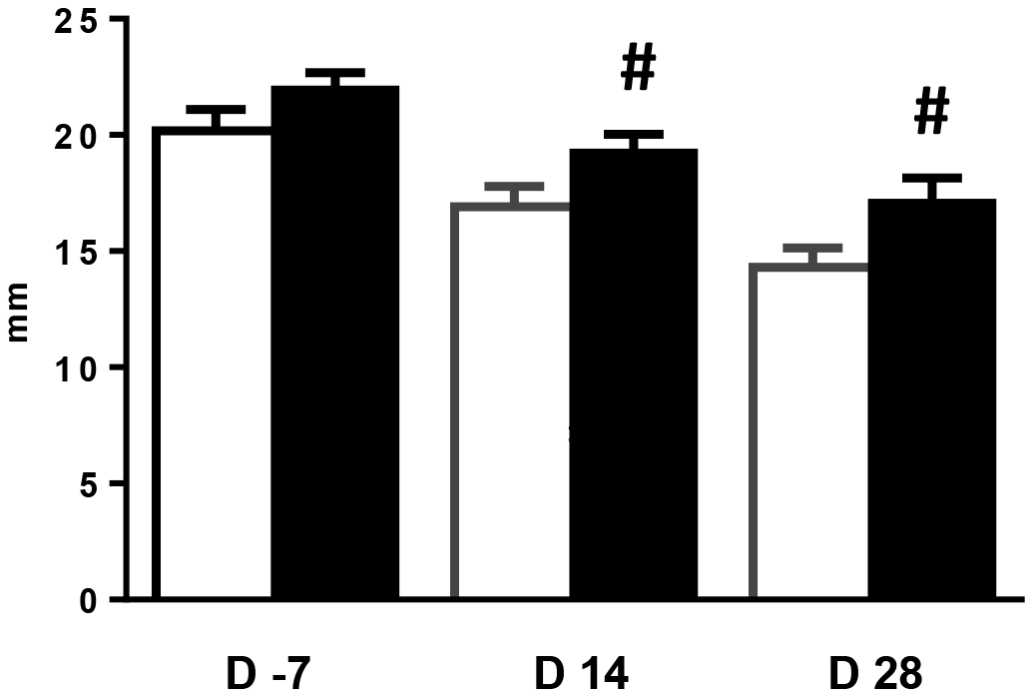
Effects of scFOS on sow back fat thickness during gestation and lactation. Sow back fat thickness was ultrasonically measured at 7 d before and 14 and 28 d after parturition in CTRL (open bars) and scFOS (filled bars) groups. Bars are mean values ± SEM; n = 16 at d -7; n = 17 at d 14 and d 28; # tendency to be different between the two groups, 0.05<*P*≤0.1.

Supplementation of the maternal diet with scFOS at the end of gestation and during lactation had no effect on sow body weight (270.5±8.7 and 271.5±8.7 kg at d 108 of gestation, 248.3±10.3 and 251.4±9.0 kg at d 14 postpartum, and 235.8±9.8 and 241.7±9.3 kg at d 28 postpartum in CTRL and scFOS groups, respectively). Variation of sow body weight from d 80 of gestation to d 28 postpartum tended to be lower in scFOS group (reduction of 11.7±2.3 and 4.7±2.9 kg in CTRL and scFOS groups, respectively; *P* = 0.07). Daily feed intake increased for the last week of lactation in sows supplemented with scFOS (no refusal during gestation in both groups; 7.7±0.2 and 8.0±0.2 kg/d in CTRL and scFOS groups respectively during the fourth week of lactation; *P*<0.05). In addition, sow back fat thickness tended to be higher in the scFOS group at d 14 (*P* = 0.06) and d 28 (*P* = 0.06) of lactation without difference before parturition **(** **Figure 2** **)**. Litter parameters were not affected by scFOS supplementation except for a tendency to a lower number of stillborn with maternal scFOS diet **(Table S3)**. This reduction was related to the small size of two scFOS litters (3 and 7 piglets).

### Colostrum and milk immune quality

**Figure 3:**
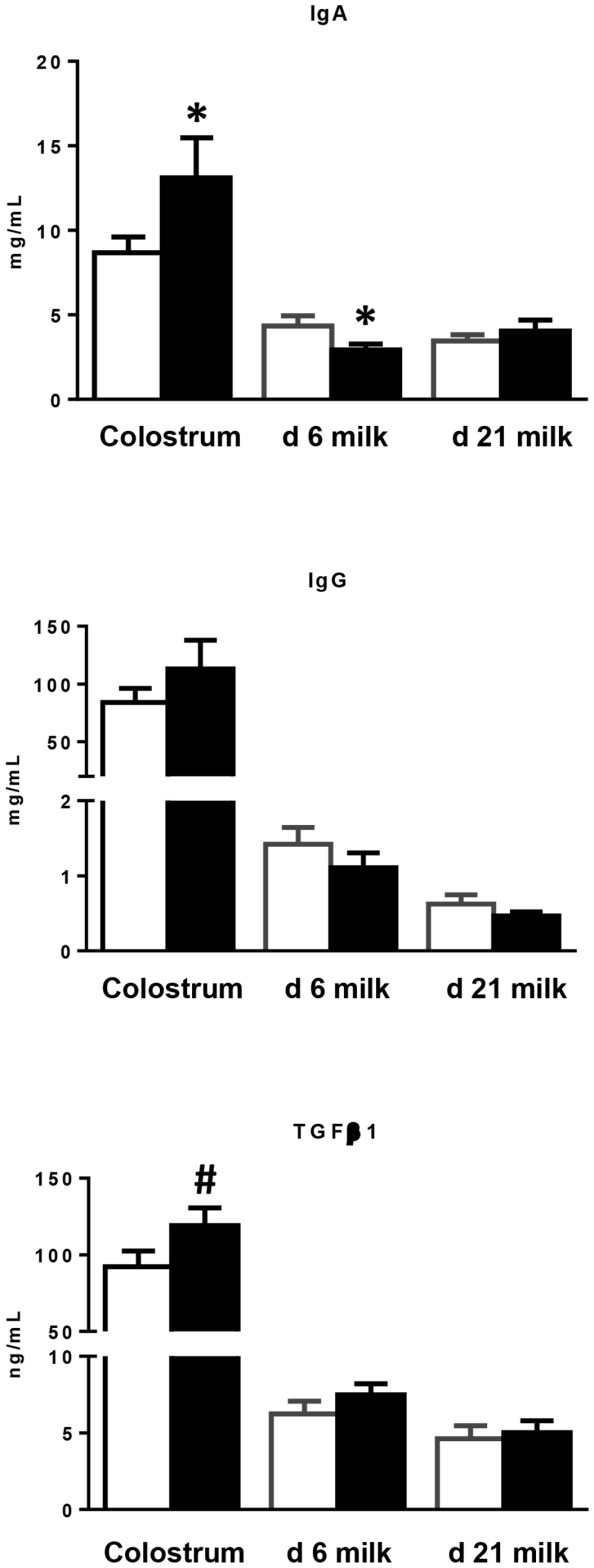
Effects of scFOS in the maternal diet on colostral and milk IgA, IgG and TGFβ1 concentrations. Concentrations of IgA, IgG and TGFβ1 in colostrum and milk of CTRL (open bars) and scFOS (filled bars) sows were analysed by ELISA assay. Bars are mean values ± SEM; n = 13–14 for colostrum; n = 17 for milks. * Significantly different between both groups, *P*≤0.05; # tendency to be different between the two groups, 0.05<*P*≤0.1.

IgA (*P*<0.05) concentration was higher and TGFβ1 concentrations tended to increase (*P* = 0.10) in scFOS colostrum compared to CTRL **(** **Figure 3** **)** whereas the concentration of IgG did not differ. No significant differences in IgG and TGFβ1 concentrations were observed between groups in d 6 and d 21 milks. IgA concentration decreased in d 6 scFOS milk (*P*<0.01) as compared to CTRL. A higher level of IgG compared with IgA was observed in colostrum for both groups while the opposite was observed in the milk at d 6 and d 21 postpartum.

### Sow and piglet serum immunoglobulin levels

No effect of scFOS on sow IgA and IgG concentrations was observed during gestation. Sow serum IgA concentrations increased (*P*<0.001) in both groups between the 87^th^ day and the 108^th^ day of gestation from 0.6±0.1 to 1.1±0.1 mg/mL in CTRL and from 0.8±0.1 to 1.1±0.1 mg/mL in scFOS. Sow serum IgG concentration was unchanged in both groups during this period of gestation (average value of 13.8±0.9 mg/mL).

Supplementation of the maternal diet with scFOS did not change serum Ig concentrations in piglets. The decrease (*P*<0.001) in serum IgA and IgG concentrations between postnatal d 7 and d 21 was similar in the two dietary groups: IgG, 15.5±0.6 mg/mL for CTRL and 15.3±0.6 mg/mL for scFOS at postnatal d 7 (n = 105–106/group) and 7.9±0.5 mg/mL for CTRL and 8.1±0.6 mg/mL for scFOS at postnatal d 21 (n = 93–98/group); IgA, 1.4±0.1 mg/mL for CTRL and 1.3±0.1 mg/mL for scFOS at postnatal d 7 (n = 105–107) and 0.14±0.01 mg/mL for CTRL and 0.14±0.01 mg/mL for scFOS at postnatal d 21 (n = 88–98/group).

### Piglet intestinal weight and morphometry at postnatal d 21

The relative length of the small intestine and its mucosa weight did not differ between the two groups **(** **Table 2** **)**. Furthermore, there was no difference between the two groups for ileal villus and crypt sizes.

**Table 2:** Effects of scFOS in the maternal diet on piglet intestinal structure at postnatal d 21.

	CTRL	scFOS	*P* value
**Piglet body weight at slaughter, ** ***kg***	6.7±0.3	7.0±0.3	0.44
**Small intestine**			
Relative length, *m/kg body weight*	1.13±0.04	1.04±0.04	0.14
Relative mucosa weight, *g/kg body weight*	0.72±0.03	0.67±0.06	0.38
**Jejunal Peyer's patches**			
Number, *n*	8.8±0.7	8.9±0.7	0.87
Mean length, *cm*	3.0±0.1	2.5±0.1	0.07
Length relative to that of small intestine, *cm/m*	3.6±0.4	3.1±0.2	0.25
Cellular density, *10^6^cells/g tissue*	5.2±4.2	4.5±2.9	0.90
**Ileal Peyer's patch**			
Mean length, *cm*	118±5	117±6	0.70
Length relative to that of small intestine, *cm/m*	15.9±0.7	16.0±0.7	0.89
Number of follicles, *n*	41±4	39±2	0.86
Follicle area, *mm^2^*	299±12	283±17	0.54
Cellular density, *10^6^cells/g*	43±7	65±16	0.22
**Mesenteric lymph nodes**			
Cellular density, *10^6^cells/g tissue*	9.8±1.1	18.7±4	0.05

Values are means ± SEM of n = 12–13.

CTRL: control diet; scFOS: maternal diet supplemented with scFOS.

Length and mucosa weight of the small intestine were reported to piglet body weight to obtain relative length and relative mucosa weight.

### Piglet PP and MLN mononuclear cell density at postnatal d 21

Although the mean length of jejunal PP tended to be lower in the scFOS group (*P* = 0.07), the total number of PP and their cell density were similar between the two groups **(** **Table 2** **)**. No differences in the length and cellular density of ileal PP were observed. In contrast, the mononuclear cell density in MLN was greater in scFOS as compared to CTRL group (*P* = 0.05; **Table 2**).

### Piglet PP and MLN cytokines, IgA secretory activity of PP cells and phenotype of ileal PP cells at postnatal d 21

**Figure 4:**
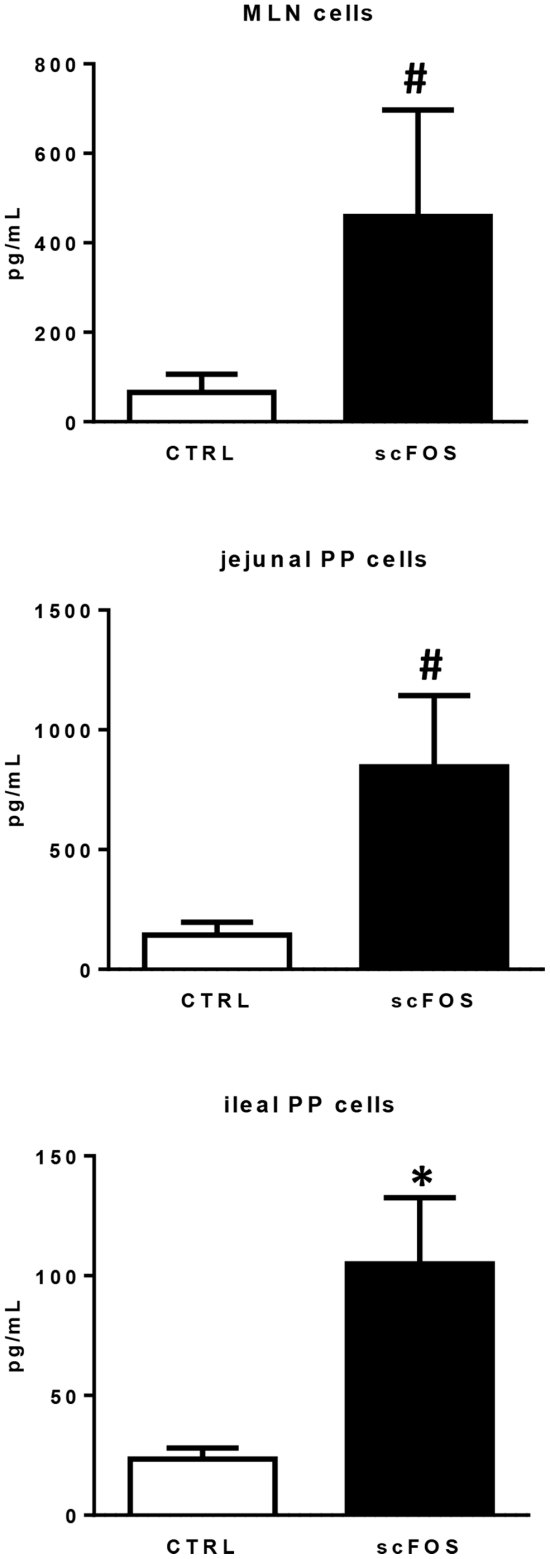
Effect of scFOS in the maternal diet on IFNγ secretion of mononuclear cells isolated from d 21 piglet MLN and jejunal and ileal PP cells. MLN and PP cells from CTRL (open bars) and scFOS (filled bars) groups were cultured *in vitro* for 72 h with or without 5 µg/mL ConA. Supernatants were harvested and ELISA was carried out to assess IFNγ secretion. Levels of IFNγ measured in basal condition were subtracted from the ones in stimulated conditions. Bars are mean values ± SEM; n = 8–11 for MLN cells; n = 3–5 for jejunal PP cells; n = 13–14 for ileal PP cells. * Significantly different between both groups, *P*≤0.05; # tendency to be different between the two groups, 0.05<*P*≤0.1.

**Figure 5:**
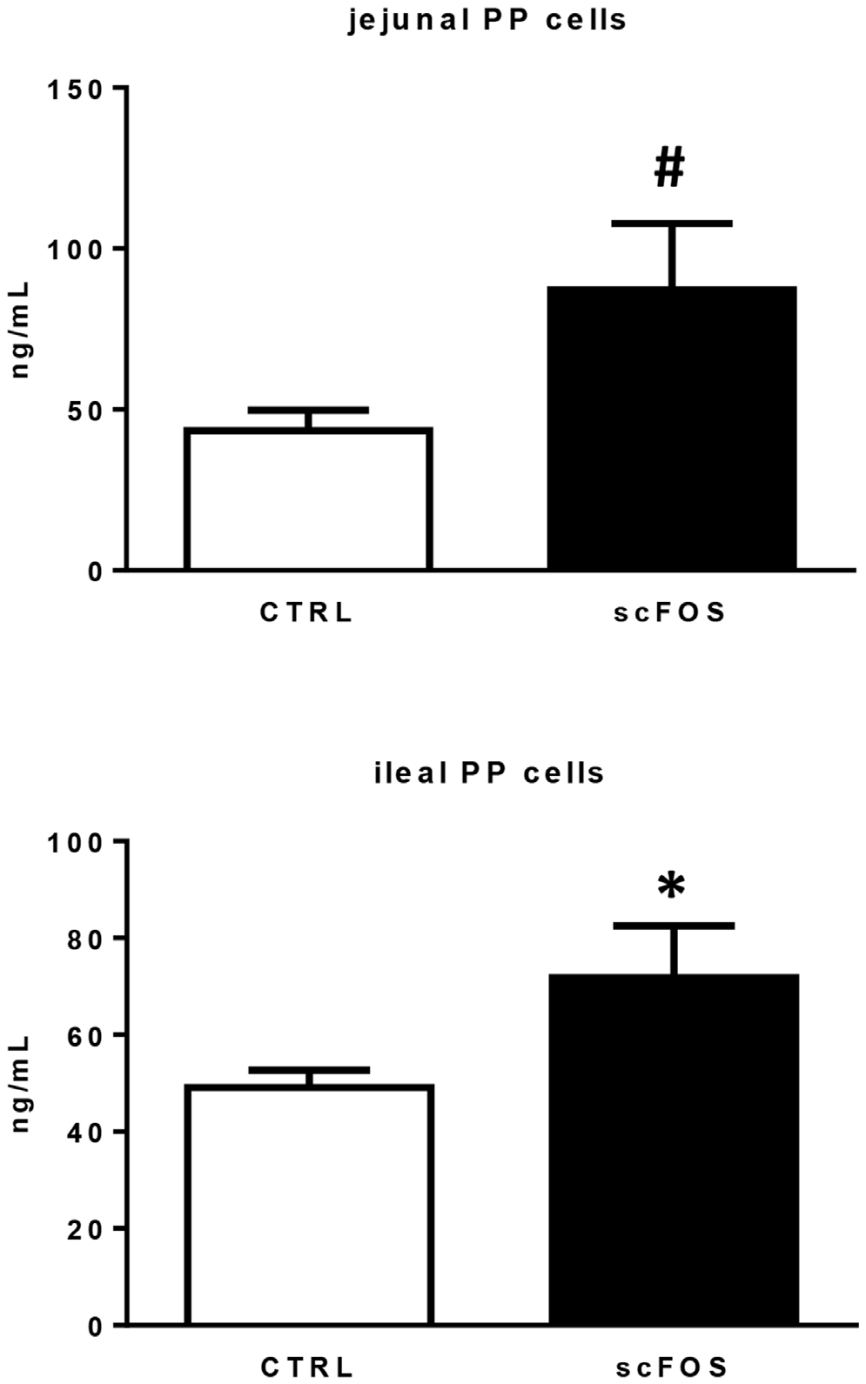
Effect of scFOS in the maternal diet on sIgA production by d 21 piglet jejunal and ileal PP cells. Culture supernatants of jejunal and ileal PP of CTRL (open bars) and scFOS (filled bars) groups were analysed by ELISA assay to determine concentration of sIgA. Data shown are mean values ± SEM; n = 5–6 for jejunal PP cells; n = 15–16 for ileal PP cells. * Significantly different between both groups, *P*≤0.05; # tendency to be different between the two groups, 0.05<*P*≤0.1.

IFNγ secretion by 72 h ConA-stimulated cells isolated from jejunal and ileal PP and MLN was increased in the scFOS group **(** **Figure 4** **)** while TNFα and IL-10 secretion was not changed with the dietary treatment (data not shown). Secretion of sIgA by 7 d cultured PP cells was significantly higher in ileum (*P*<0.05) and tended to be higher in jejunum (*P*<0.10) of the scFOS group **(** **Figure 5** **)**. There were no significant differences regarding cytokine secretions in unstimulated conditions (data not shown).

To decipher the functional modifications of ileal PP cells isolated from scFOS *vs*. CTRL piglets, we analysed by flow cytometry the cell phenotype before culture **(** **Table 3** **)**. The proportion of B cells Ig+CD21+ was similar in both groups, although sIgA secretion *in vitro* was greater in the scFOS group. The proportion of MHC class II+ antigen-presenting cells, the mean fluorescence intensity, particularly that of MHC class II+CD172α+CD11R1- cells (ileal PP dendritic cells) and MHC class II+CD14+ (monocytes/macrophages), did not vary. The proportion of helper T cells, cytotoxic T cells, double positive T cells, natural killer cells, γδ T cells and regulatory T cells was not affected by the dietary treatment. The proportion of activated CD25+CD4α+ T cells among the CD4+ helper T lymphocytes was significantly higher in the scFOS group (86.5±5.6% for scFOS and 66.7±4.3% for CTRL; *P*<0.05). Moreover, such proportion of activated CD25+CD4α+ T cells was positively correlated with IFNγ secretion of ileal PP cells (P<0.05, Pearson R square = 0.29).

**Table 3:** Effects of scFOS in the maternal diet on phenotype of ileal PP cells isolated from piglets at postnatal d 21.

	CTRL	scFOS	*P* value
**% of total viable cells**			
B cells Ig+CD21+*(%)*	24.69±2.76	22.17±2.95	0.56
MHC class II+ *(%)*	38.29±4.41	43.72±5.47	0.45
MHC class II+CD172+CD11R1−*(%)*	0.94±0.24	2.02±0.77	0.15
MHC class II+CD14+ *(%)*	0.77±0.21	0.97±0.25	0.53
Helper T cells CD8−CD2+CD4+ *(%)*	2.37±0.54	1.61±0.37	0.31
Activated T cells CD25+CD4+ *(%)*	1.60±0.27	1.25±0.30	0.41
Reg T cells FoxP3+CD4+ *(%)*	0.43±0.11	0.39±0.19	0.86
Cytotoxic T cells CD8+CD2+CD4−*(%)*	0.67±0.18	0.53±0.14	0.58
Double positive T cells CD8+CD2+CD4+ *(%)*	0.13±0.04	0.24±0.08	0.20
Natural Killer cells CD8+CD16+CD4−*(%)*	0.44±0.12	0.18±0.06	0.11
γδ T cells TCR1+ *(%)*	0.51±0.15	0.43±0.14	0.72
**% of CD4+ helper T cells**			
Activated T cells CD25+CD4α+	66.7±4.3	86.5±5.6	0.01

Cells were analysed with a MACSQuant analyser (Miltenyi Biotech, Paris, France) equipped with MACSQuantify software. Data are mean values of cell percentage ± SEM; n = 7–11.

CTRL: control diet; scFOS: maternal diet supplemented with scFOS.

### Piglet plasma haptoglobin at d 21

**Figure 6:**
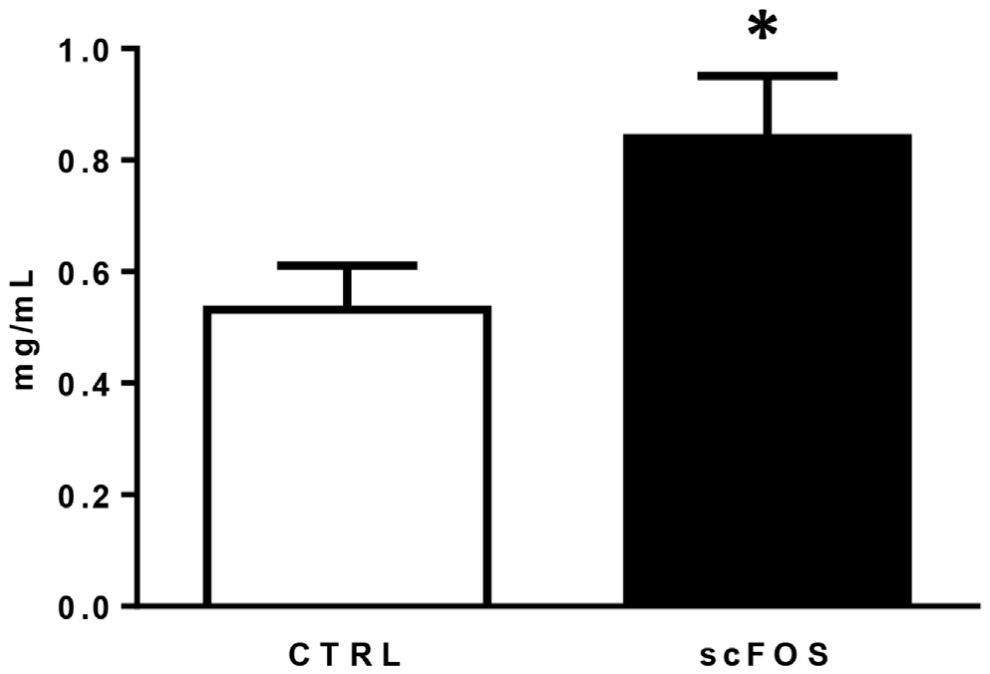
Effect of scFOS in the maternal diet on d 21 piglet plasma haptoglobin in CTRL (open bar) and scFOS (filled bar) groups. Data are mean values ± SEM; n = 128–131. * Significantly different between the two groups, *P*≤0.05.

The level of plasma haptoglobin was greater in scFOS (+58%, *P*<0.05) compared to CTRL group, though values corresponded to normal physiological levels in both groups (<1 mg/mL) **(** **Figure 6** **)**.

### Dynamic profile of SCFA concentration in piglet digesta at postnatal d 10, d 21, d 49 and d 90

**Figure 7:**
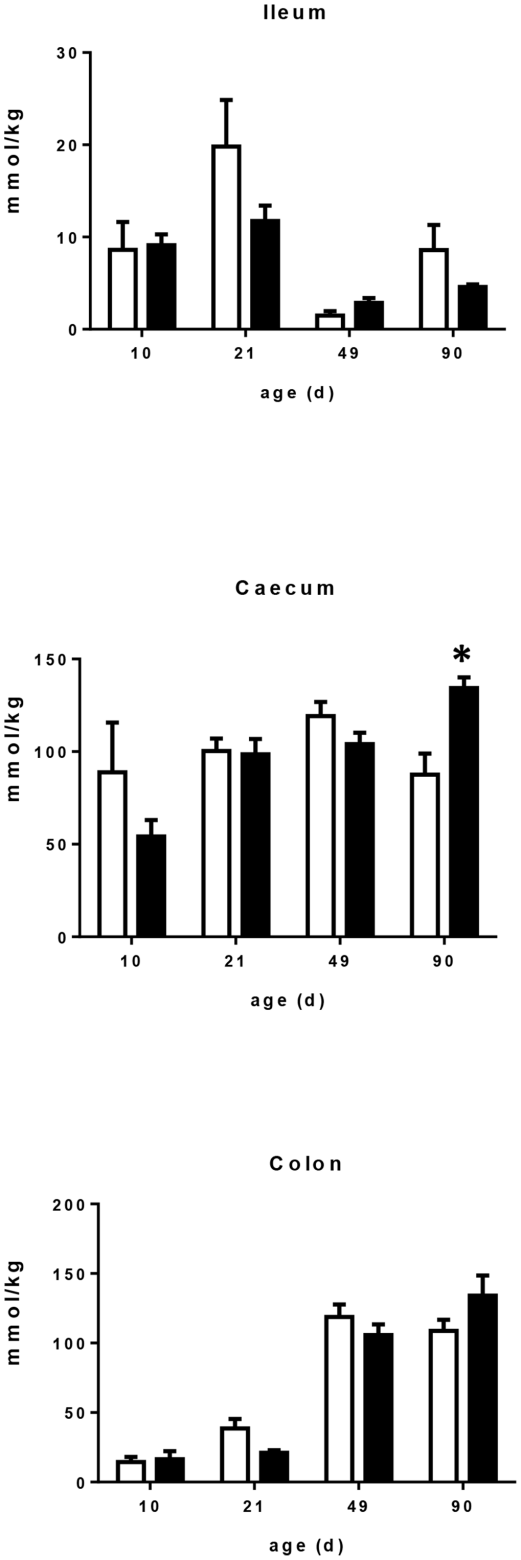
Effect of scFOS in the maternal diet on total SCFA in ileum, caecum and colon of CTRL (open bars) and scFOS (filled bars) groups. Data are mean values ± SEM; n = 4. * Significantly different between the two groups, *P*≤0.05.

Concentration of total SCFA changed significantly throughout the experimental period in all intestinal segments **(** **Figure 7** **)**. Ileal SCFA concentration decreased sharply after weaning (*P*<0.001) but was not affected by the maternal dietary treatment. In the colon, SCFA concentration increased after weaning whatever the maternal dietary treatment. In the caecum it was unchanged from postnatal d 10 up to d 90 in the CTRL group. However it was higher at postnatal d 49 and d 90 compared to postnatal d 10 in the scFOS group (*P*<0.05), resulting in higher content of SCFA in scFOS than CTRL group at postnatal d 90 (*P* = 0.05). Caecal content of postnatal d 90 scFOS piglets contained more propionate, butyrate and valerate (*P*<0.05, *P* = 0.06 and *P*<0.03, respectively) **(Figure S1)** while branched-chain fatty acid (isobutyrate and isovalerate) contents were not affected by the dietary treatment.

## Discussion

Our study demonstrated that supplementing the maternal diet with scFOS modified the development of mucosal cellular immunity in suckling offspring: increased secretion of pro-inflammatory cytokines by PP and MLN cells, higher proportion of activated T-cells in ileal PP cells and increased secretion of sIgA by PP cells. This effect was associated with improved lactogenic immunity and with modification of the fermentative activity of caecal microbiota.

### Maternal dietary scFOS leads to a lower mobilization of sow body reserves during lactation

Maternal scFOS supplementation did not induce any change in body weight of piglets. The effect of maternal prebiotic supplementation on offspring birth weight and postnatal growth is controversial with increased neonatal body weight in pigs and mice [27], [34], decreased neonatal body weight in dogs [26] or unchanged neonatal body weight in human [22]. The tendency to a lesser reduction in back fat thickness of scFOS sows during lactation in our study may be explained by an improved insulin sensitivity in scFOS animals since scFOS have been described to improve insulin sensitivity in obese insulin-resistant animals [35], [36]. Similarly in our study, scFOS may have reduced the insulin resistance that is currently observed in sows during late gestation and lactation periods [37]. The tendency to a lesser reduction in sow body weight during the experimental period and a higher fat mass with scFOS supplementation during lactation could improve the reproductive performances of sows when their body reserves are less mobilized [38].

### Maternal dietary scFOS improved lactogenic immunity

Colostral IgA and TGFβ1 concentrations were increased when the maternal diet was supplemented with scFOS during the last 4 weeks of gestation. Gourbeyre et al. have reported higher IgA and TGFβ1 concentrations in the milk of mice fed a 4% GOS-enriched diet during gestation and lactation [28]. A *peripartum* supplementation of sow diets with MOS (8 g/d) also resulted in higher colostral IgA and IgG concentrations [27]. The increase in colostral IgA was not concomitant with a change in IgA levels in sow serum, suggesting that it is due to a higher rate of synthesis in the immunological sites including the mammary gland with reference to the immune entero-mammary axis [39].

There have been considerable advances in the understanding of milk components that influence the immune status of infants. Protection against intestinal pathogens is partly conferred by milk-derived immunity until weaning. This so-called lactogenic immunity is associated with sIgA, predominant in mucosal surfaces [3]. The resistance of Ig towards gut digestion gives the milk-derived sIgA a first-class role in the intestinal defence against microbial antigens. sIgA limit the adherence and invasion of potentially harmful antigens in mucosal tissues and neutralize toxins and virulence factors from bacterial pathogens [40]. In addition to the IgA increase, the greater concentration of colostral TGFβ1, locally produced by the mammary gland [41], influences the maturation and development of immune cells in offspring [42]. TGFβ1 has been reported to both improve the acquisition of tolerance to dietary antigens when orally administered [43] and to play a role in isotype switching to IgA. TGFβ1 cooperates with CD40 ligand, a tumour necrosis factor (TNF) family member expressed by CD4+ T cells, to trigger IgA class-switch DNA recombination and generate antigen-specific IgA+ B cells [44]. Therefore, the high colostral IgA and cytokine concentration induced by scFOS may modify the early process of commensal microbiota establishment in the intestine and of sIgA responses needed to maintain gut homeostasis [45].

### Maternal dietary scFOS accelerated intestinal immune system development

We examined whether maternal dietary scFOS influenced the intestinal immunity of the offspring. We focused on PP and MLN mononuclear cells as they represent inductive sites of the intestinal immune response. In line with the absence of prebiotics in the intestinal lumen of piglets, we observed no change in the size of non-immunological (villus height and crypt depth) and immunological (size and number of PP follicles) structures. This should be contrasted with the trophic effect mostly reported with orally-fed prebiotics on villous size as well as on PP cellular density and follicle size [13], [17], [18]. It is noteworthy that the cellular density in MLN was greater in piglets from scFOS supplemented sows, which could be associated with a better or accelerated homing of immune cells when development of intestinal immunity occurred. The balance between Th1 and Th2 responses in the newborn is skewed towards a Th2 profile, resulting in high susceptibility to intracellular pathogen infection [46]. Previous studies have demonstrated that neonates are not able to mount an efficient immune response to a large number of pathogens because Th1 responses are compromised at several steps including the deficient production of Th1 type cytokines by CD4+ T cells and mononuclear phagocytes [47]. The impaired protection of the neonate against infections has been attributed to a deficient secretion of IFNγ [48], [49]. Therefore higher IFNγ secretion in our study may indicate that maternal scFOS administration produced conditions for polarization of Th1 responses by PP and MLN cells. Flow cytometry analysis of ileal PP cells demonstrated a higher proportion of activated CD25+CD4α+ T lymphocytes among helper T cells in scFOS piglets compared to CTRL piglets. The amount of activated CD25+CD4α+ T cells was positively correlated with IFNγ secretion. Overall, these results revealed an activation of CD4+ T cells and accelerated pro-Th1 polarization after maternal scFOS supplementation. Accordingly, the level of plasmatic haptoglobin in d 21 piglets was increased following maternal scFOS supplementation. This inflammatory marker has been described as being associated with stimulation of Th1 cellular response by increasing IFNγ [50].

In addition, increased sIgA production by jejunal and ileal PP cells was observed in piglets whose mothers were fed scFOS. Similarly, sIgA secretion by PP cells was improved with scFOS intake in rodents [18], [32]. In our study, we did not show different proportions of Ig+CD21+ B cells in ileal PP cells, in agreement with Nakamura et al. [32]. However, the increase in sIgA by ileal PP cells could be due to specific IgA+ B cells [32].

### Maternal dietary scFOS has a mid-term effect on the fermentative activity of offspring microbiota

To gain insight into the effects of maternal scFOS supplementation on the piglets, we postulated that scFOS induced changes in the microbiota of pregnant sows and that this modified microbiota was transmitted to the piglets at birth, as is well documented in the literature [23], [24], [51]. To assess potential modifications, we evaluated the bacterial fermentative activity by analysing SCFA concentration in intestinal segments of suckling (postnatal d 10 and d 21) and weaned (postnatal d 49 and d 90) piglets. No change was observed in the bacterial metabolite profile of ileal, caecal and colonic contents in suckling piglets, perhaps because at that stage there was no fibre to be fermented in the distal intestine. However, when the microbiota of scFOS piglets was exposed to a standard fibre-rich dietary environment after weaning, it was able to produce significantly more SCFA than that of CTRL piglets. These results revealed a delayed modification of microbiota activity in offspring due to scFOS-supplemented maternal diet, with a similar impact on SCFA production to that observed with mice early exposed to prebiotics via maternal supplementation during the perinatal period and feed supplementation at weaning [28], [52]. In our study, SCFA did not explain changes in the ileal immune system development, as there were no changes in intestinal SCFA concentrations of suckling piglets. However the modulation of the bacterial metabolite profile observed in older pigs may reveal a different microbiota, which may have been different from the suckling period. Maternal scFOS supplemented diet-induced changes in offspring microbiota composition warrants further investigations.

Such a different composition of the microbiota early in life would have influenced immune cell polarization by antigen-presenting cells, in particular dendritic cells. The nature of T cell-polarizing signals is determined largely by the type of microbial products [53]. Studies demonstrated that *Bifidobacterium* components, known to be increased with prebiotic supplementation [8], were effective in improving IFNγ secretion in T cells [54] *via* polarization of DC towards Th1 cell responses [55]. Moreover, colonization with *Lactobacilli*, increased with prebiotic incorporation in the diet as well, improved the numbers of IgA+ B cells in intestinal mucosa [56]. In our study, the increased sIgA secretion following dietary scFOS supplementation could be explained by the increase in IFNγ secretion. Indeed, it has been shown that IFNγ increased the expression of pIgR receptor that could improve the transport of the polymeric form of IgA across the epithelium to the mucosal surface [40].

In conclusion, perinatal supplementation of scFOS was shown to stimulate the passive immunity transferred to the offspring and to impact the development and maturation of the mucosal immune system in suckling piglets. Maternal supplementation with scFOS was associated with modification in Th1 response with more activated T cells, positively correlated to IFNγ secretion, and a higher production of sIgA by PP cells. Such results underline the key role of perinatal nutrition in supporting early development of the mucosal immune system. The nutritional environment of the gestating female must be considered with caution considering the defence capacity of the offspring. The long-term consequences of these early changes for adult health are unknown and warrant further investigations.

## Supporting Information

Figure S1 **Effect of scFOS in the maternal diet on SCFAs in caecal contents of CTRL (open bar) and scFOS (filled bar) groups.** Data are mean values ± SEM; n = 4. * Significantly different between the two groups, P≤0.05; # tendency to be different between the two groups, 0.05<P≤0.1.
(TIF)

Table S1 **Dilutions used for IgA and IgG assays.**
(DOCX)

Table S2 **List of antibodies and the corresponding cell types recognized.**
(TIF)

Table S3 **Effects of scFOS in the maternal diet on litter size and alive piglet body weight.**
(TIF)
