# Supplementary figures and images for: Maternal Short-Chain Fructooligosaccharide Supplementation Influences Intestinal Immune System Maturation in Piglets
Source: PLoS One. 2014 Sep 19;9(9):e107508. doi: 10.1371/journal.pone.0107508 (PMC4169551; doi:10.1371/journal.pone.0107508)

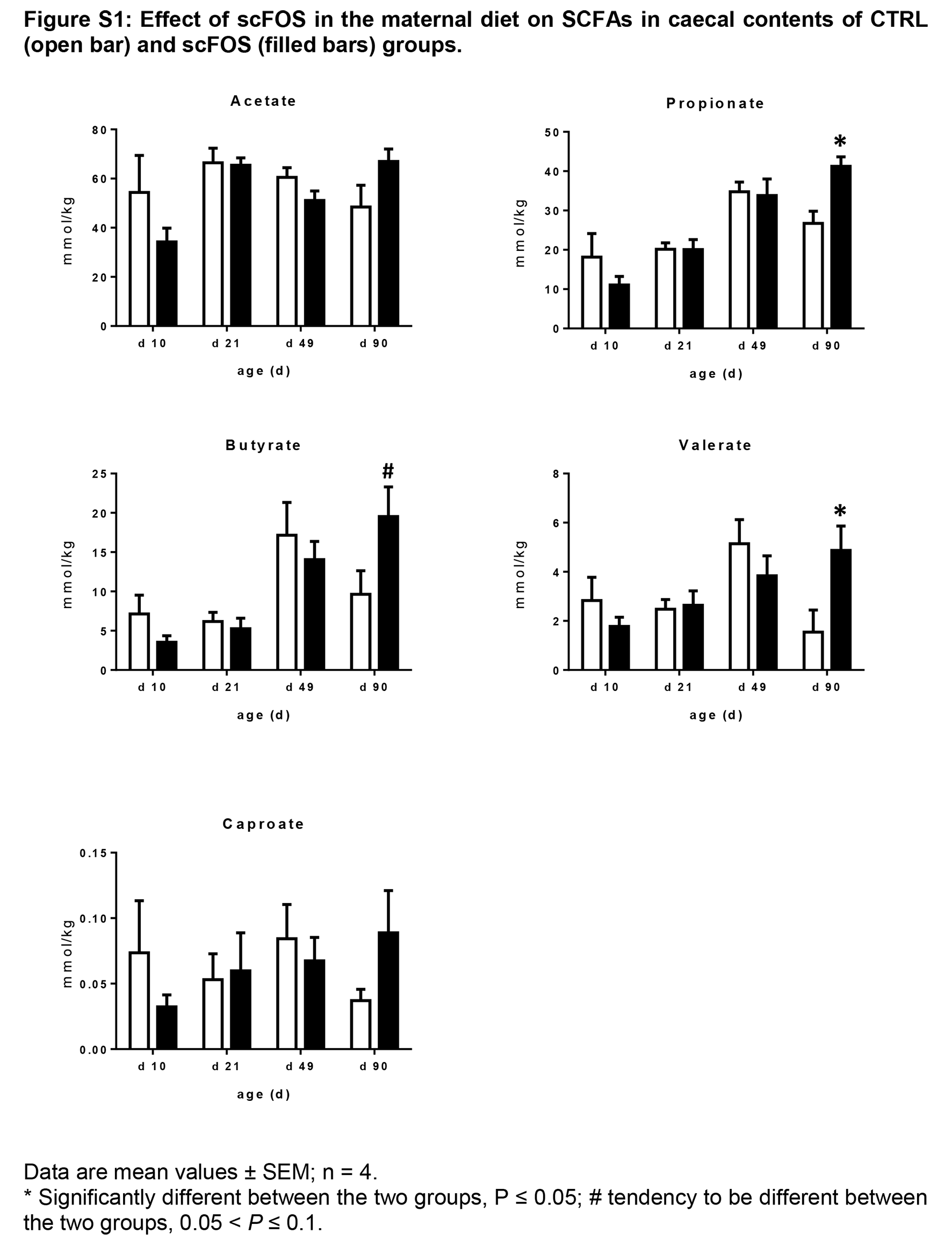

Supplement: Figure S1 — Effect of scFOS in the maternal diet on SCFAs in caecal contents of CTRL (open bar) and scFOS (filled bar) groups. Data are mean values ± SEM; n = 4. * Significantly different between the two groups, P≤0.05; # tendency to be different between the two groups, 0.05<P≤0.1. (TIF) [file pone.0107508.s001.tif]

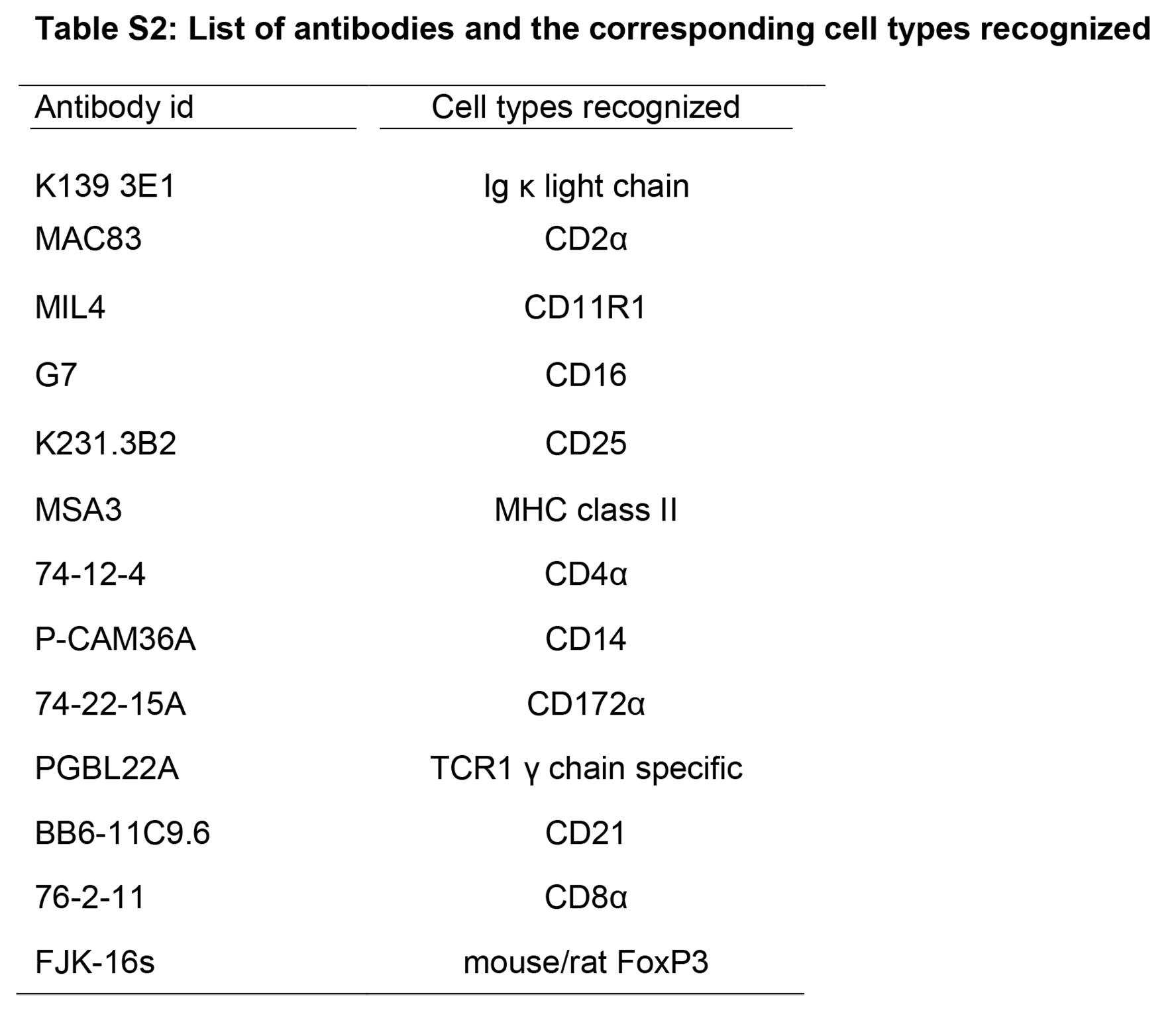

Supplement: Table S2 — List of antibodies and the corresponding cell types recognized. (TIF) [file pone.0107508.s003.tif]

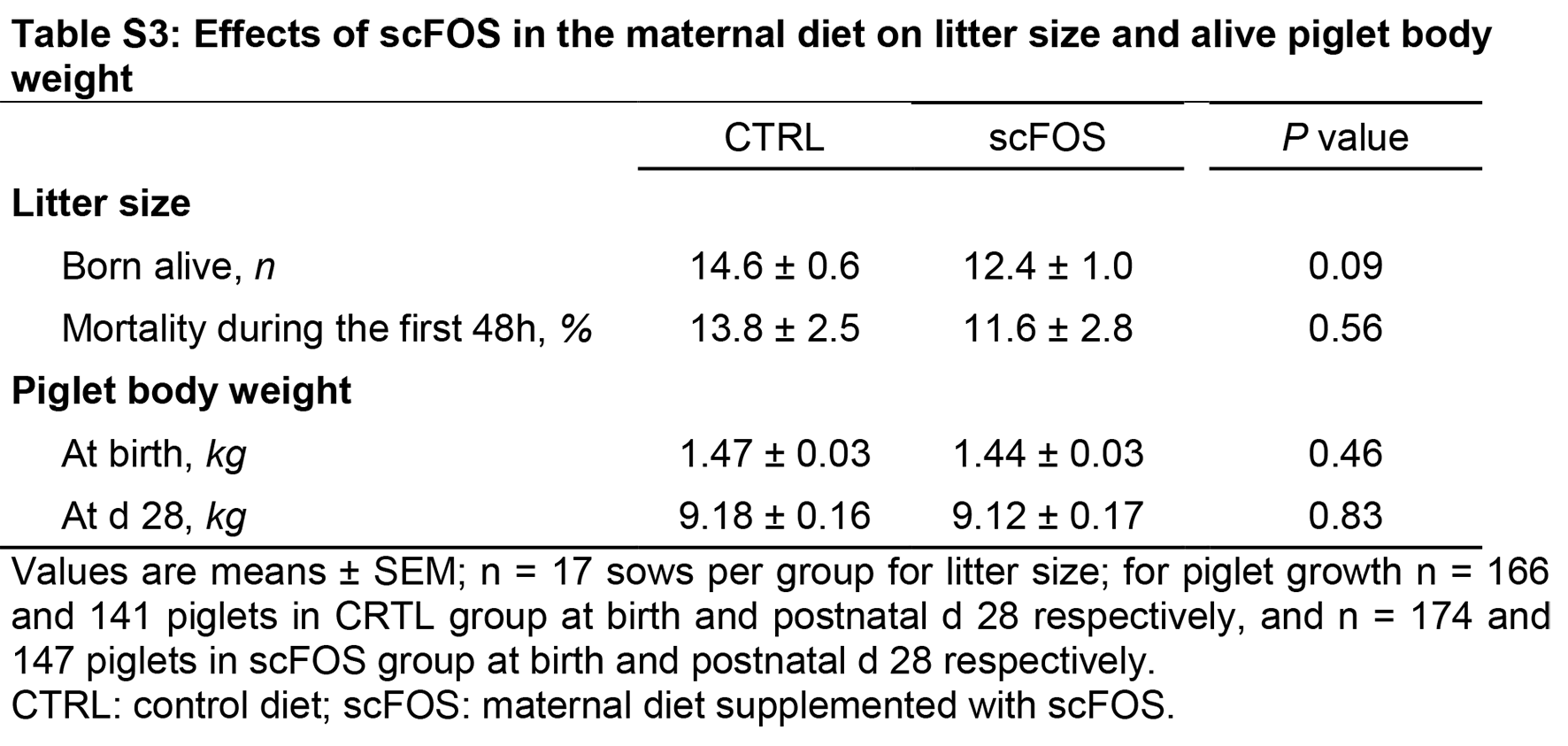

Supplement: Table S3 — Effects of scFOS in the maternal diet on litter size and alive piglet body weight. (TIF) [file pone.0107508.s004.tif]
