# Supplementary material for: Maternal Short-Chain Fructooligosaccharide Supplementation Influences Intestinal Immune System Maturation in Piglets
Source: PLoS One. 2014 Sep 19;9(9):e107508. doi: 10.1371/journal.pone.0107508 (PMC4169551; doi:10.1371/journal.pone.0107508)
Supplement: Table S1 — Dilutions used for IgA and IgG assays. (DOCX) [file pone.0107508.s002.docx]

**Table S1: Dilutions used for IgA and IgG assays**

|  | **IgA** | **IgG** |
| --- | --- | --- |
| **Sample** | **Dilution** | **Dilution** |
| D 28 and d 7 sow serum before parturition | 1/3,000 | 1/100,000 |
| D 7 piglet serum | 1/15,000 | 1/100,000 |
| D 21 piglet serum | 1/3,000 | 1/100,000 |
| Colostrum | 1/100,000 | 1/250,000 |
| d 6 and d 21 milk | 1/25,000 | 1/10,000 |
| Cultured Peyer’s patches supernatant d 21 | 1 | / |
